# Efficient production of anthocyanins in *Saccharomyces cerevisiae* by introducing anthocyanin transporter and knocking out endogenous degrading enzymes

**DOI:** 10.3389/fbioe.2022.899182

**Published:** 2022-08-19

**Authors:** Sha Xu, Guangjian Li, Jingwen Zhou, Guicai Chen, Jianzhong Shao

**Affiliations:** ^1^ College of Life Sciences, Key Laboratory for Cell and Gene Engineering of Zhejiang Province, Zhejiang University, Hangzhou, China; ^2^ National Engineering Laboratory for Cereal Fermentation Technology, Jiangnan University, Wuxi, China; ^3^ Zhejiang Esigma Biotechnology Company Limited, Haining, China; ^4^ Laboratory for Marine Biology and Biotechnology, Qingdao National Laboratory for Marine Science and Technology, Qingdao, China

**Keywords:** anthocyanins, degrading enzyme, *Saccharomyces cerevisiae*, transporter, *de novo* biosynthesis

## Abstract

Anthocyanins are natural pigments found in various plants. As multifunctional natural compounds, anthocyanins are widely used in food, pharmaceuticals, health products, and cosmetics. At present, the anthocyanins are heterologously biosynthesized in prokaryotes from flavan-3-ols, which is rather expensive. This study aimed to metabolically engineer *Saccharomyces cerevisiae* for anthocyanin production. Anthocyanin production has been extensively studied to understand the metabolic pathway enzymes in their natural hosts, including CHS (chalcone synthase); FLS (flavonol synthase); CHI (chalcone isomerase); F3H (flavanone 3-hydroxylase); F3′H (flavonoid 3′-hydroxylase); F3′5′H (flavonoid 3′,5′-hydroxylase); DFR (dihydroflavonol 4-reductase); ANS (anthocyanidin synthase); LAR (leucoanthocyanidin reductase); and UFGT (flavonoid 3-O-glucosyltransferase). The anthocyanin transporter *Md*GSTF6 was first introduced and proven to be indispensable for the biosynthesis of anthocyanins. By expressing *Md*GSTF6, *Fa*DFR, *Ph*ANS_0_, and *Dc*3GT and disrupting *EXG1* (the main anthocyanin-degrading enzyme), the BA-22 strain produced 261.6 mg/L (254.5 mg/L cyanidin-3-*O*-glucoside and 7.1 mg/L delphinidin-3-*O*-glucoside) anthocyanins from 2.0 g/L dihydroflavonols, which was known to be the highest titer in eukaryotes. Finally, 15.1 mg/L anthocyanins was obtained from glucose by expressing the *de novo* biosynthesis pathway in *S. cerevisiae*, which is known to be the highest *de novo* production. It is the first study to show that through the introduction of a plant anthocyanin transporter and knockout of a yeast endogenous anthocyanin degrading enzyme, the anthocyanin titer has been increased by more than 100 times.

## Introduction

Anthocyanins are water-soluble pigments belonging to the class of flavonoids existing in a plethora of plants, which endow nature with rich colors (Khoo et al., 2017). They are mainly stored in the vacuoles of plant cells (Liu et al., 2018). More than 700 anthocyanins have been verified (Salehi et al., 2020), among which pelargonidin, cyanidin, delphinidin, peonidin, petunidin, and malvidin are the most common types (Khoo et al., 2017). They play important roles in plant growth by protecting against pathogens and reducing the harmful effects of ultraviolet radiation (Jiang et al., 2019). A large number of studies have demonstrated that anthocyanins have multiple physiological activities within the human body, such as improvement in vision (Nomi et al., 2019), prevention of brain disorders (Henriques et al., 2020), intervention against obesity (Yamei et al., 2020), and treatment of hyperglycemia and hyperuricemia (Yang et al., 2020). As natural multifunctional additives, anthocyanins are approved as food colorants by many countries (Belwal et al., 2020). Given that the market demand for anthocyanins is growing, more diverse strategies for improving the efficiencies of anthocyanin production are yet to be explored. However, issues such as the “browning effect,” (Oszmianski and Lee, 1990), unstable supply, and low efficiency remain to be resolved.

**FIGURE 1:**
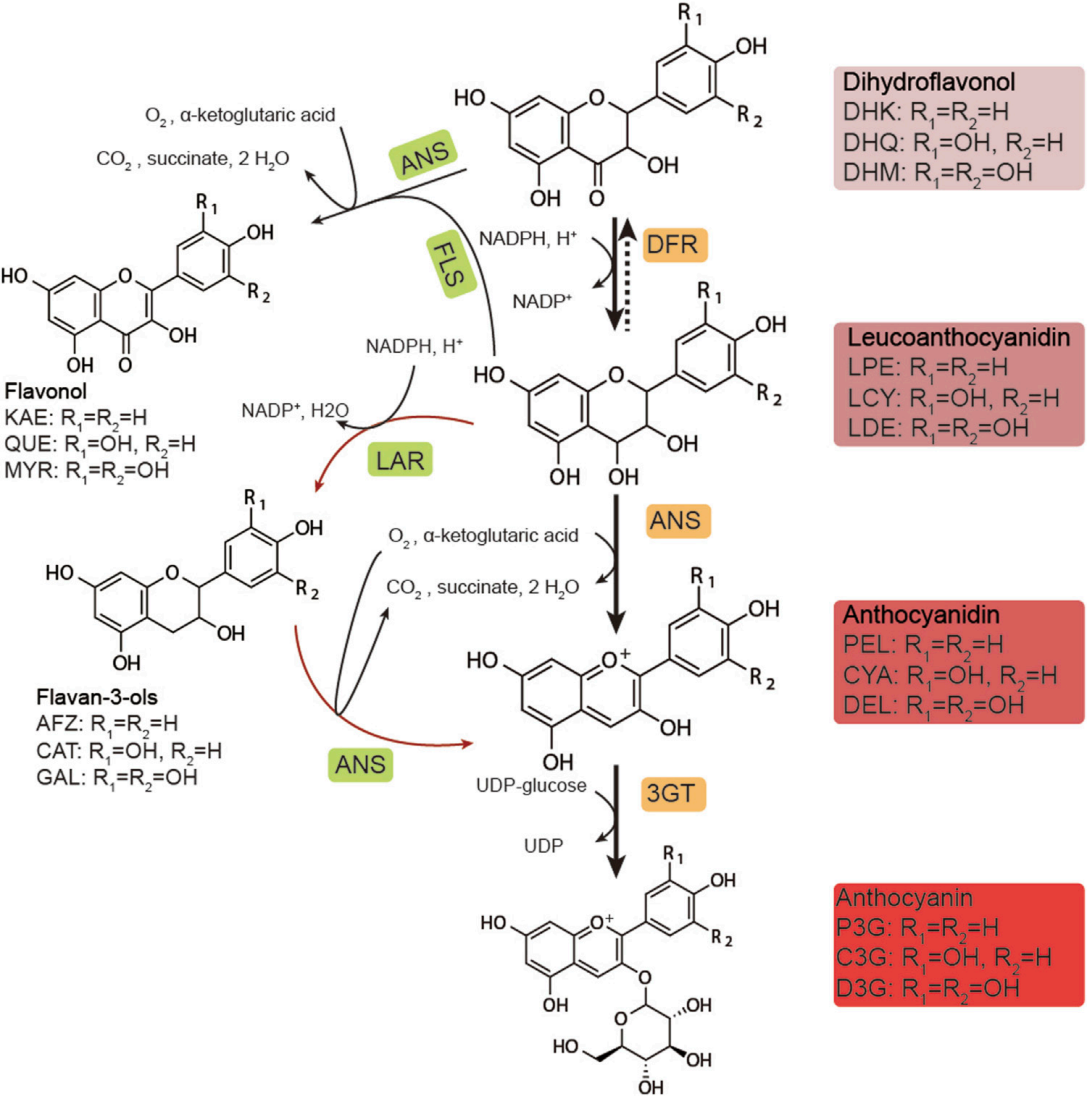
Biosynthetic pathway of anthocyanins. DHK, dihydrokaempferol; DHQ, dihydroquercetin; DHM, dihydromyricetin; LPE, leucopelargonidin; LCY, leucocyanidin; LDE: leucodelphinidin; PEL, pelargonidin; CYA, cyanidin; DEL, delphinidin; P3G, pelargonidin-3-*O*-glucoside; C3G, cyanidin-3-*O*-glucoside; D3G, delphinidin-3-*O*-glucoside; KAE, kaempferol; QUE, quercetin; MYR, myricetin; AFZ, afzelechin; CAT, catechin; GAL, gallocatechin; DFR, dihydroflavonol-4-reductase; ANS, anthocyanidin synthase; 3GT, UDP-glucose flavonoid 3-*O*-glucosyltransferase; FLS, flavonol synthase; LAR, leucoanthocyanidin reductase.

Multiple genes are involved in the biosynthesis of anthocyanins (Ichino et al., 2014). In previous studies, synthesis commenced from naringenin (NAR) and proceeded through a series of hydroxylation reactions catalyzed by flavonoid 3-hydroxylase (F3H), flavonoid 3′-hydroxylase (F3′H), and flavonoid 3′5′-hydroxylase (F3′5′H) (Figure 1). These dihydroflavonols were reduced to unstable intermediates; leucoanthocyanidins by dihydroflavonol 4-reductase (DFR) (Belwal et al., 2020; Saigo et al., 2020). These were converted into anthocyanidins in the form of aglycone by anthocyanidin synthase (ANS) and then subsequently converted into anthocyanin in the form of glycoside by UDP-glucose flavonoid 3-*O*-glucosyltransferase (UFGT/3GT). The biosynthesis of anthocyanin in plants has been extensively studied (Passeri et al., 2016) and is strictly regulated by the MBW (MYB-bHLH-WDR) protein complex in plant cells (Xu et al., 2015). After synthesis in the cytoplasm, the anthocyanins are known to be transported into the vacuole and restored (Chanoca et al., 2016; Olejnik et al., 2016; Jiang et al., 2019).

Like most high-value natural compounds, the acquisition of anthocyanins still relies significantly on extractions from plants. The slow growth of plants and their dependence on arable land, as well as the long-winded, destructive extraction methods, makes high-value chemicals so expensive (Belwal et al., 2020; Marchev et al., 2020). Using microbes is an alternative and benign approach for anthocyanin production (Yan et al., 2005). The heterologous biosynthesis of anthocyanins was first reported in *Escherichia coli*, and titers of up to 439 mg/L of cyanidin-3-*O*-glucoside (C3G) from catechin have been obtained in this microbial host (Yan et al., 2008; Lim et al., 2015; Shrestha et al., 2019). Alternative prokaryotic hosts such as *Corynebacterium glutamicum* (Zha et al., 2018) and *Lactococcus lactis* (Solopova et al., 2019) have also proven themselves as capable hosts for anthocyanin production, though in these particular cases, flavan-3-ols (afzelechin and catechin) were used as the initial starting point, which was rather expensive. However, inefficient conversion of dihydroflavonols to anthocyanins remains unresolved*.* In this work*, Saccharomyces cerevisiae* was engineered to biosynthesize anthocyanins by introducing an anthocyanin transporter, screening of optimal genes, and identifying as well as knock out of endogenous anthocyanin endogenous degrading enzymes. Finally, 261.6 mg/L and 15.1 mg/L anthocyanins were obtained from dihydroflavonols and glucose, respectively.

## Materials and methods

### Genes, plasmids, strains, and chemicals

All the genes, plasmids, and strains used in this study are listed in Supplementary Tables S1, S2 and Table 1. The genes were synthesized by Sangon (Shanghai, China), which were optimized for *S. cerevisiae* expression, except for *Gh*ANS_0_, *Ph*ANS_0_, and *At*3GT_0_. The pY26-TEF-GPD plasmid (Li et al., 2008) was used for gene expression, and the pRS426-P_TEF1_-Cas9-P_SNR52_-sgRNA plasmid was used for genome editing of *S. cerevisiae*. *E. coli* JM109 was used for plasmid propagation.

**TABLE 1:** Strains used in this study.

Strain	Relevant property
CENPK2-1D	*MATα; ura3-52; trp1-289; leu2-3,112; his3Δ1; MAL2-8* ^*C*^ *; SUC2*
BA-01	*gal80::P*_*GAL7*_ *-At3GT*, CENPK2-1D
BA-02	*gal80::P*_*GAL7*_ *-At3GT*_*0*_, CENPK2-1D
BA-03	*gal80::P*_*GAL7*_ *-Fa3GT*, CENPK2-1D
BA-04	*gal80::P*_*GAL7*_ *-Dc3GT*, CENPK2-1D
BA-05	BA-04 harboring the pY26-P_TDH1_-*Fa*DFR-T_GAA1_-P_GAL10_-G*h*ANS_0_-T_ALT1_-P_INO1_-*Md*GSTF6-T_CYC1_ plasmid
BA-10	*SCW2Δ*, CENPK2-1D
BA-11	*SIM1Δ*, CENPK2-1D
BA-12	*SCW4Δ*, CENPK2-1D
BA-13	*EXG1Δ*, CENPK2-1D
BA-14	*SPR1Δ*, CENPK2-1D
BA-15	*YIR007WΔ*, CENPK2-1D
BA-16	*SIM1Δ*, BA-10
BA-17	*SCW4Δ*, BA-16
BA-18	*EXG1Δ*, BA-17
BA-19	*SPR1Δ*, BA-18
BA-20	*YIR007WΔ*, BA-19
BA-21	*gal80::P*_*GAL7*_ *-Dc3GT*, BA-20
BA-22	BA-21 harboring the pY26-P_TDH1_-*Fa*DFR-T_GAA1_-P_GAL10_-*Gh*ANS_0_-T_ALT1_-P_INO1_-*Md*GSTF6-T_CYC1_ plasmid
BA-23	BA-21 harboring the pY26-P_TDH1_-*Fa*DFR-T_GAA1_-P_GAL10_-*Ph*ANS-T_ALT1_ plasmid
BA-24	pY26-P_TDH1_-*Fa*DFR-(GGGGS)2-*Vv*LAR-T_GAA1_-P_GAL10_-*Ts*ANS-T_ALT1_-P_INO1_-*Md*GSTF6-T_CYC1_
BM-31	Obtained in previous study Li et al. (2020a)
BA-31	*exg1::P*_*GAL7*_ *-Dc3GT*, BM-31
BA-32	BA-31 harboring the pY26-P_TDH1_-*Fa*DFR-T_GAA1_-P_GAL10_-*Ph*ANS_0_-T_ALT1_-P_INO1_-*Md*GSTF6-T_CYC1_ plasmid

The endogenous genes of yeast are generally shown in italics.

Cyanidin, cyanidin-3-*O*-glucoside, delphinidin, and delphinidin-3-*O*-glucoside were purchased from Herbest (Baoji, China). Catechin, protocatechuic acid, and phloroglucinol aldehyde were purchased from Yuanye (Shanghai, China). The BM-31 strain was used for dihydroflavonol production in a 5-L bioreactor following the protocol of culture conditions described in Section 2.5. The substrate used in this study was obtained through methanol extraction and concentration using an RE100-Pro rotary evaporator (DLAB Scientific, Beijing, China). The substrate contained 69.3% of dihydroquercetin, 24.0% of dihydromyricetin, and 6.7% of other intermediate products.

### Genetic procedures

All the DNA fragments were amplified using 2 × Phanta Max Master Mix (Dye Plus) (Vazyme, Nanjing, China). A ready-to-use seamless cloning kit (Sangon, Shanghai, China) was used for plasmid construction; the length of the homologous arm was not less than 25 bp between the fragments. All the recombinant plasmids were determined by DNA sequencing. The knockout and integration of genes were performed using CRISPR-Cas9 systems (Dicarlo et al., 2013). The CRISPRdirect tool (http://crispr.dbcls.jp) was used for selecting a 20-nt guide sequence of the single-guide RNA (sgRNA). All the 20-nt guide sequences used in this study are listed in Supplementary Table S3. The gene deletion and expression cassettes (donor DNA) for genome editing were obtained by overlap extension polymerase chain reaction, in which the homologous arms were amplified from the genome and were not less than 300 bp. A Frozen-EZ Yeast Transformation II Kit (Zymo Research, CA, United States) was used for the transformation of *S. cerevisiae* following the manufacturer’s protocol.

### Ammonium sulfate precipitation

The strain CENPK2-1D was grown on the YPD medium (10 g/L yeast extract, 20 g/L tryptone, and 20 g/L glucose) plate for 2–3 days at 30°C. Five to seven colonies were inoculated into a 250-ml shaking flask containing 20 ml of the YPD medium for 16 h and cultivated at 220 rpm and 30°C. Four 2-L shaking flasks containing 500 ml of YPD medium were inoculated with 5 ml of preculture for 24-h cultivation at 220 rpm and 30°C. Then, the culture was centrifuged at 4°C and 4000 g for 10 min using an Avanti J-26S XP Centrifuge (Beckman Coulter, CA, United States). Approximately 2 L of the supernatant was collected in a 4-L centrifuge bucket and placed on a magnetic stirrer in an ice bath. Then, 328 g (NH_4_)_2_SO_4_ was added slowly to the supernatant with stirring, which made the concentration of ammonium sulfate in the solution reach 30% (g/g), followed by centrifugation at 4°C and 4,000 g for 20 min. The supernatant was collected into a centrifuge bucket again; 10 ml 20 mmol/L PB (phosphate buffer, pH = 5.0, 4°C) was used to dissolve the protein precipitate, and 0–30% protein component was obtained. Then, 234 g (NH_4_)_2_SO_4_ was slowly added to the supernatant mentioned above with stirring, which made the concentration of ammonium sulfate in the solution reach 50% (g/g), followed by centrifugation at 4°C and 4,000 g for 20 min; the supernatant was collected into the centrifuge bucket again, 10 ml of 20 mmol/L PB was used to dissolve the protein precipitate, and 3–50% protein component was obtained. Finally, 696 g (NH_4_)_2_SO_4_ was added slowly to the supernatant with stirring, which made the concentration of ammonium sulfate in the solution reach 100% (g/g), followed by centrifugation at 4°C and 4,000 g for 20 min, the supernatant was removed, and 10 ml of 20 mmol/L PB was used to dissolve the protein precipitate, and 50–100% of the protein component was obtained.

### Identification of anthocyanin-degrading enzymes

The protein component was filtered through a 0.45-μm filter membrane before purification. ÄKTA Avant 25 (GE Healthcare, WA, United States) equipped with HiLoad 26/600 Superdex 200 pg (GE Healthcare) was used for size-exclusion chromatography. The protein component was loaded at the rate of 5 ml/min, and 20 mm Tris, pH 5.0 was used for elution at the rate of 3 ml/min. The eluates corresponding to different signal peaks were collected. Amicon Ultra Centrifugal Filters of 10k MWCO (Merck, NJ, United States) were used for the protein concentration at 3000 g and 4°C until the volume was concentrated to 200 μl. SDS-PAGE was used for separating the protein concentrates using a NuPAGE 10% Bis-Tris Gel (Thermo Fisher Scientific, MA, United States). The protein bands were excised from the gel. The protein profiles provided by Sangon (Shanghai, China) were used for identifying the protein bands.

### Preparation of dihydroflavonols

Dihydroflavonols were produced from glucose by the strain of BM-31 (Li G. et al., 2020) through the fed-batch fermentation as described below with a modification that supplemented 10 g/L CaCO_3_ in the medium. The fermentation broth was placed in a 4-L centrifuge bucket and then centrifuged for 15 min at 4°C and 1,500 g using an Avanti J-26S XP (Beckman Coulter, CA, United States). The supernatant and cell pellet were treated separately: the water from the supernatant was removed by using a rotary evaporator, an equal volume of ethanol was added to the solid, and suspension A was obtained; equal volume of ethanol was added to the cell pellet, and suspension B was obtained. Suspensions A and B were mixed, followed by magnetic stirring for 24 h to extract flavonoids, then placed in a Buchner funnel for suction filtration, and washed with an appropriate amount of ethanol. The filtrate was evaporated to dryness using a rotary evaporator; finally, a small amount of ethanol was used to dissolve precipitated flavonoids. The components of dihydroflavonols in the extract were 95%, of which DHQ (dihydroquercetin) and DHM (dihydromyricetin) account for 72 and 23%, respectively, as per the analysis by HPLC.

### Culture conditions

All the recombinant yeasts were grown on the auxotrophic SC medium (1.7 g/L yeast nitrogen base, 5.0 g/L ammonium sulfate, 20 g/L glucose, and appropriate amino acids: 50 mg/L leucine, 50 mg/L histidine, 50 mg/L tryptophan, and 50 mg/L uracil) plate for 2–4 days at 30°C. A single colony was inoculated into a 12-ml sterile culture tube containing 2 ml of SC medium for 24-h growth at 220 rpm and 30°C. Then, 0.5 ml of preculture was inoculated into a 250-ml shake flask containing 50 ml of SC medium at 220 rpm and 30°C. After 24-h growth, the culture was centrifuged at 4°C and 3000 g for 5 min using an Allegra X-15R Centrifuge (Beckman Coulter, CA, United States). Then, the strains were resuspended in 10 ml of YPD medium supplemented with 500 mg/L dihydroflavonols for 24-h cultivation.

The fed-batch fermentation was performed, and dissolved oxygen was maintained at 10% (degree of saturation) with the agitation ranging from 250 to 600 rpm. A 5-L bioreactor (T&J Bioengineering, Shanghai, China) contained 2.5 L of YPD medium for *de novo* biosynthesis of anthocyanins. Medium A (400 g/L glucose, 18 g/L KH_2_PO_4_, 15 g/L yeast extract, 10.24 g/L MgSO_4_7H_2_O, 7 g/L K_2_SO_4_, 0.56 g/L Na_2_SO_4_, 20 ml/L trace metal solution, and 24 ml/L vitamin solution) was as described in the previous study (Li G. et al., 2020); it fed for 15 h at the rate of 5 ml/h and stopped at 60 h. After 24 h, 0.05 mmol/L α-ketoglutaric acid, 0.5 mmol/L ascorbic acid, and 0.5 mmol/L FeSO_4_ were added individually to the bioreactor.

### Analytical methods

Synergy H1 (BioTek, VT, United States) was used for measuring OD_600_. Equal volumes of fermentation broth and acidified methanol (1% HCL, *v*/*v*) were mixed for cell disruption using FastPrep-24 5^G^ (MP Biomedicals, Shanghai, China). Then, the mixed liquid was centrifuged and filtered using a 0.22-μm filter membrane. A high-performance liquid chromatography system (Shimadzu Corporation, Kyoto, Japan) equipped with a diode array detector was used for flavonoid analysis (Li G. et al., 2020). Cyanidin, cyanidin-3*-O-*glucoside, delphinidin, delphinidin-3*-O-*glucoside, and catechin were detected at 527 nm, 517 nm, 532 nm, 524 nm, and 278 nm, respectively. Dihydroquercetin, dihydromyricetin, protocatechuic acid, and phloroglucinol aldehyde were detected at 290 nm.

## Results

### Introduction of an anthocyanin vacuolar transporter

**FIGURE 2:**
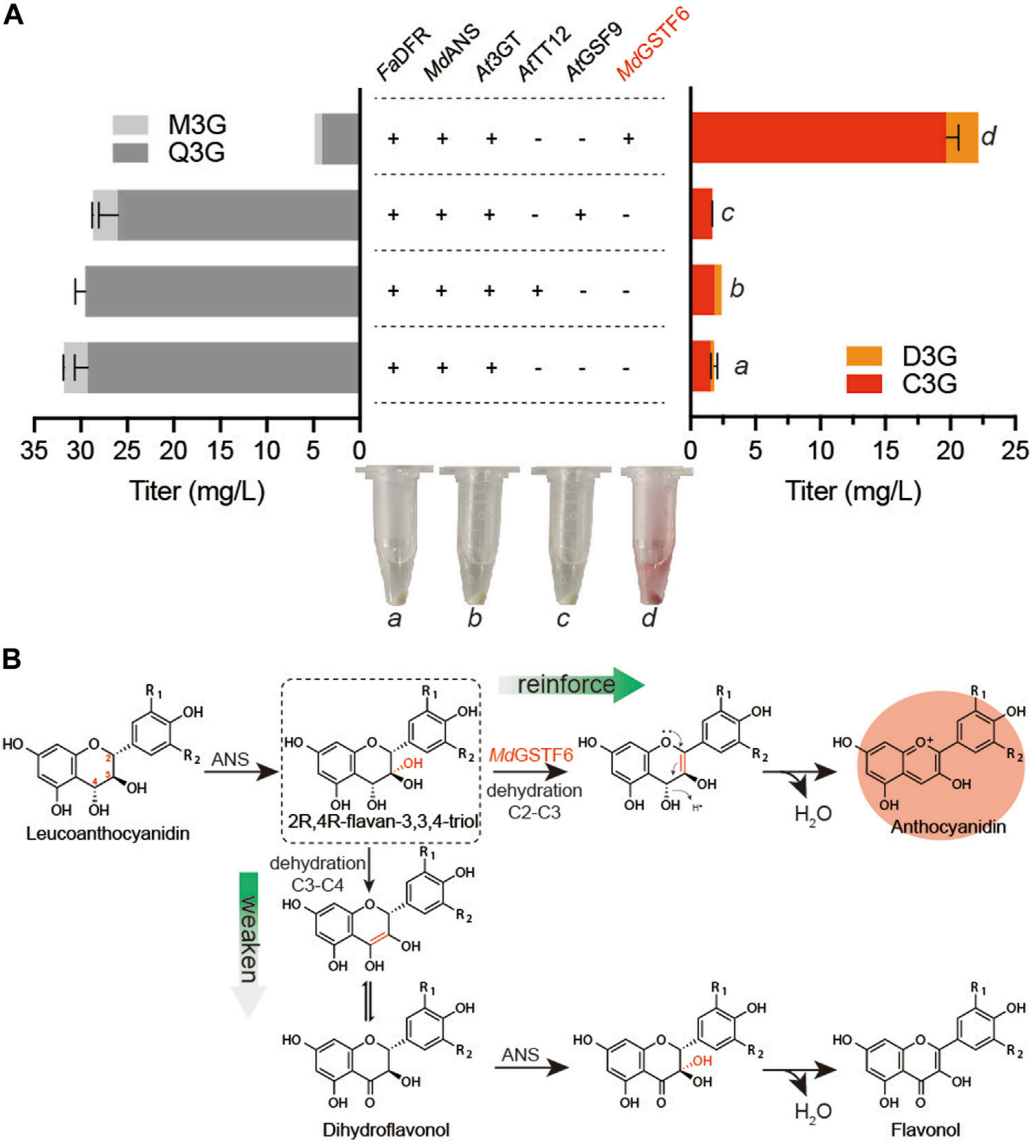
Metabolic engineering strategies for anthocyanin production **(A)** *At*TT12, *At*GSF9, and *Md*GSTF6 promoted by P_INO1_ co-expressed with *Fa*DFR and *Md*ANS promoted by P_TDH1_ and P_GAL10_ in the BA-01 strain with the pY26-TEF-GPD. The “a,” “b,” “c,” and “d” are the fermentation broth (equal volume of acidified methanol was added) of different groups **(B)** Possible mechanism of *Md*GSTF6 for anthocyanin production in *S. cerevisiae*.

As a eukaryote, *S. cerevisiae* has been proven to be a suitable host for the biosynthesis of many natural products, such as artemisinic acid (Paddon et al., 2013). It has organelles similar to plant cells, especially the vacuole that is also the storehouse in plant cells. Some transporters of anthocyanin from plant sources were reported to promote vacuolar absorption of anthocyanins when expressed in *S. cerevisiae* (Zhao and Dixon, 2009). Therefore, three transporters, *At*TT12 (Zhao and Dixon, 2009), *At*GFS9 (Ichino et al., 2014), and *Md*GSTF6 (Jiang et al., 2019), were synthesized and co-expressed with *Fa*DFR, *Md*ANS, and *At*3GT (Supplementary Table S1), respectively. Supplemented with 500 mg/L dihydroflavonols for 24-h cultivation, the results are shown in Figure 2A When *Fa*DFR, *Md*ANS, and *At*3GT were expressed in *S. cerevisiae*, only 1.8 mg/L anthocyanins (1.6 mg/L C3G and 0.2 mg/L D3G) were obtained. However, 22.1 mg/L anthocyanins (19.6 mg/L C3G and 2.5 mg/L D3G) were obtained when *Md*GSTF6 was co-expressed in the aforementioned strain, which was greater than that of *At*TT12 and *At*GFS9. Meanwhile, ANS belonging to the 2-oxoglutarate-dependent dioxygenase (2ODD), has properties of another 2ODD flavanol synthase (FLS) that catalyzes the conversion of dihydroflavonols into flavonols (Wilmouth et al., 2002); then, it can be glycosylated by 3GT (Lyu et al., 2020). Therefore, quercetin-3-*O*-glucoside (Q3G) and myricetin-3-*O*-glucoside (M3G) were detected in this study. The introduction of *Md*GSTF6, on the contrary, significantly reduced the occurrence of side reactions; the contents of flavonol glycosides were decreased to 4.9 mg/L (4.1 mg/L Q3G and 0.8 mg/L M3G).

### Screening of high-efficiency anthocyanidin synthase and UDP-glucose flavonoid 3-*O*-glucosyltransferase

**FIGURE 3:**
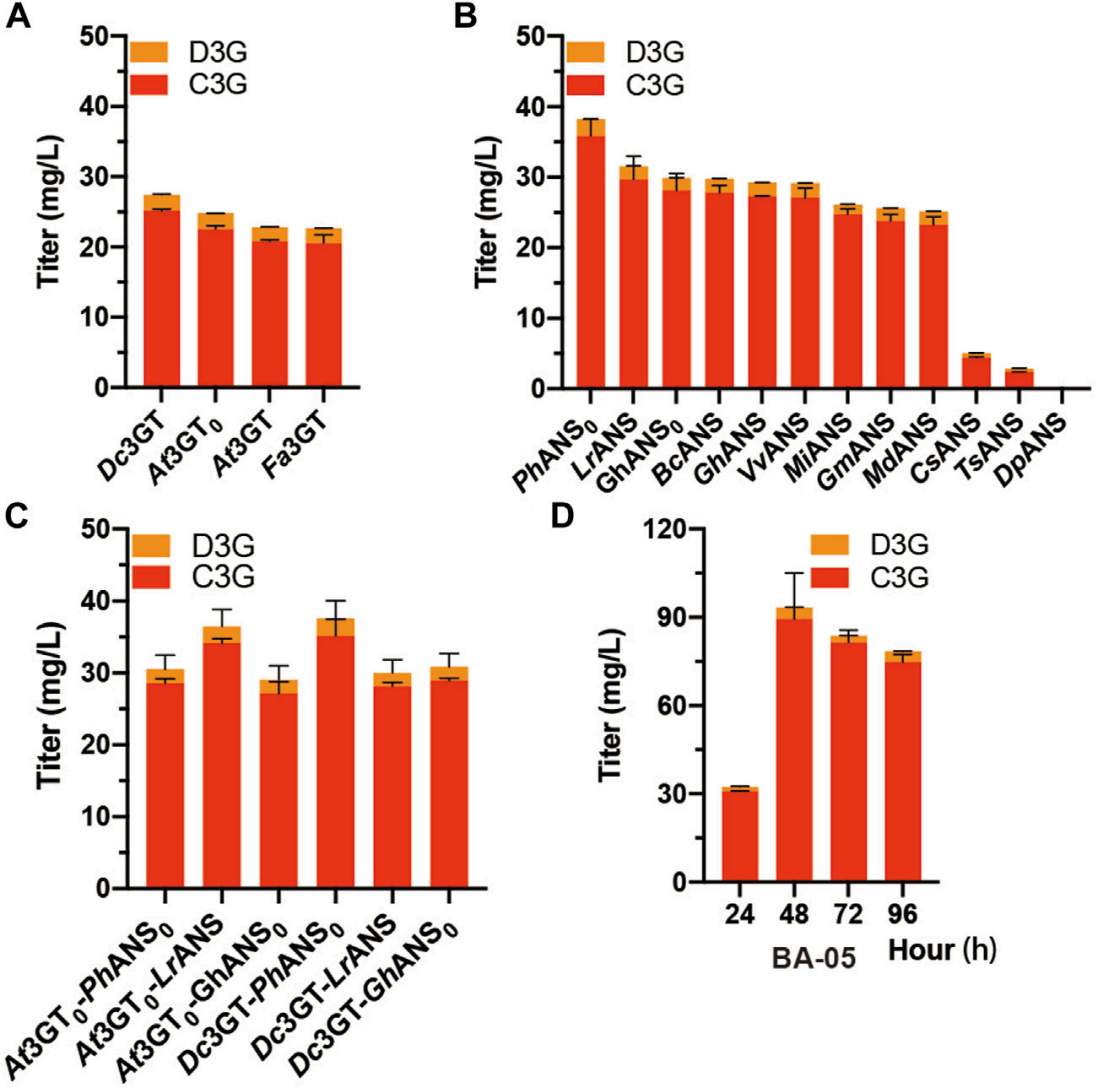
Screening of ANS and 3 GT **(A)** *At*3GT, *At*3GT_0_, *Fa*3GT, and *Dc*3GT represent BA-01, BA-02, BA-03, and BA-04, respectively, harboring the pY26-P_TDH1_-*Fa*DFR-T_GAA1_-P_GAL10_-*Md*ANS-T_ALT1_-P_INO1_-*Md*GSTF6- T_CYC1_ plasmid for 24-h cultivation supplemented with 500 mg/L dihydroflavonols **(B)** BA-04 harboring 12 different pY26-P_TDH1_-*Fa*DFR-T_GAA1_-P_GAL10_-ANSs-T_ALT1_-P_INO1_-*Md*GSTF6-T_CYC1_ plasmids (ANSs indicate 12 different types of ANS) for 24-h cultivation supplemented with 500 mg/L dihydroflavonols **(C)** Two strains BA-02 and BA-04 and three plasmids pY26-P_TDH1_-*Fa*DFR-T_GAA1_-P_GAL10_-ANSs-T_ALT1_-P_INO1_-*Md*GSTF6-T_CYC1_ plasmid (ANSs indicate *Ph*ANS_0_, *Lr*ANS, and *Gh*ANS_0_) composed of six combinations for 24-h cultivation supplemented with 500 mg/L dihydroflavonols **(D)** 96-h fermentation of the BA-05 strain; 500 mg/L dihydroflavonols were added after 0 h, 1,000 mg/L dihydroflavonols were added after 24 h, and 500 mg/L dihydroflavonols were added after 48 h.

In the anthocyanin biosynthetic process, the unstable aglycone form (anthocyanidin) can be converted into a stable glycoside form (anthocyanin) by UDP-glucose flavonoid 3-*O*-glucosyltransferase (3GT). Another three 3GTs, namely, *At*3GT_0_, *Fa*3GT, and *Dc*3GT were integrated into the *GAL80* site of CENPK2-1D, resulting in BA-02, BA-03, and BA-04 (Table 1). The pY26-P_TDH1_-*Fa*DFR-T_GAA1_-P_GAL10_-*Md*ANS-T_ALT1_-P_INO1_-*Md*GSTF6-T_CYC1_ plasmid was transformed into the strains of BA-01, BA-02, BA-03, and BA-04. After 24-h cultivation, the *Dc*3GT was optimal and 27.4 mg/L anthocyanins (25.1 mg/L C3G and 2.3 mg/L D3G) were obtained when supplemented with 500 mg/L dihydroflavonols (Figure 3A). Anthocyanidin synthase (ANS) is a vital enzyme for biosynthesis of anthocyanin. Other 11 ANS, namely, *Dp*ANS, *Gh*ANS, *Gh*ANS_0_, *Lr*ANS, *Ts*ANS, *Vv*ANS, *Bo*ANS, *Cs*ANS, *Gm*ANS, *Mi*ANS, and *Ph*ANS_0_ were tested in the strain BA-04. The result (Figure 3B) showed *Ph*ANS_0_ had the highest activity and 38.2 mg/L anthocyanins (35.8 mg/L C3G and 2.4 mg/L D3G) were obtained. After “were obtained.”, please add a sentence “Two 3GT (*At*3GT_0_, *Dc*3GT) and three ANS (*Ph*ANS_0_, *Lr*ANS, *Gh*ANS_0_) were paired into six groups that *Dc*3GT and *Ph*ANS_0_ were found to be the best catalytic combination (Figure 3C), when supplemented with 2 g/L dihydroflavonol, 83.7 mg/L (81.4 mg/L C3G and 2.3 mg/L D3G) anthocyanins were obtained at 48-h fermentation (Figure 3D).” It is worth noting that the catalytic ability of *At*3GT and *At3*GT_0_, *Gh*ANS, and *Gh*ANS_0_ was higher than that of their codon-optimized counterparts. These results indicate that *S. cerevisiae* can be used as a host for the expression of enzymes from plant sources.

### Identification of endogenous anthocyanin-degrading enzymes

**FIGURE 4:**
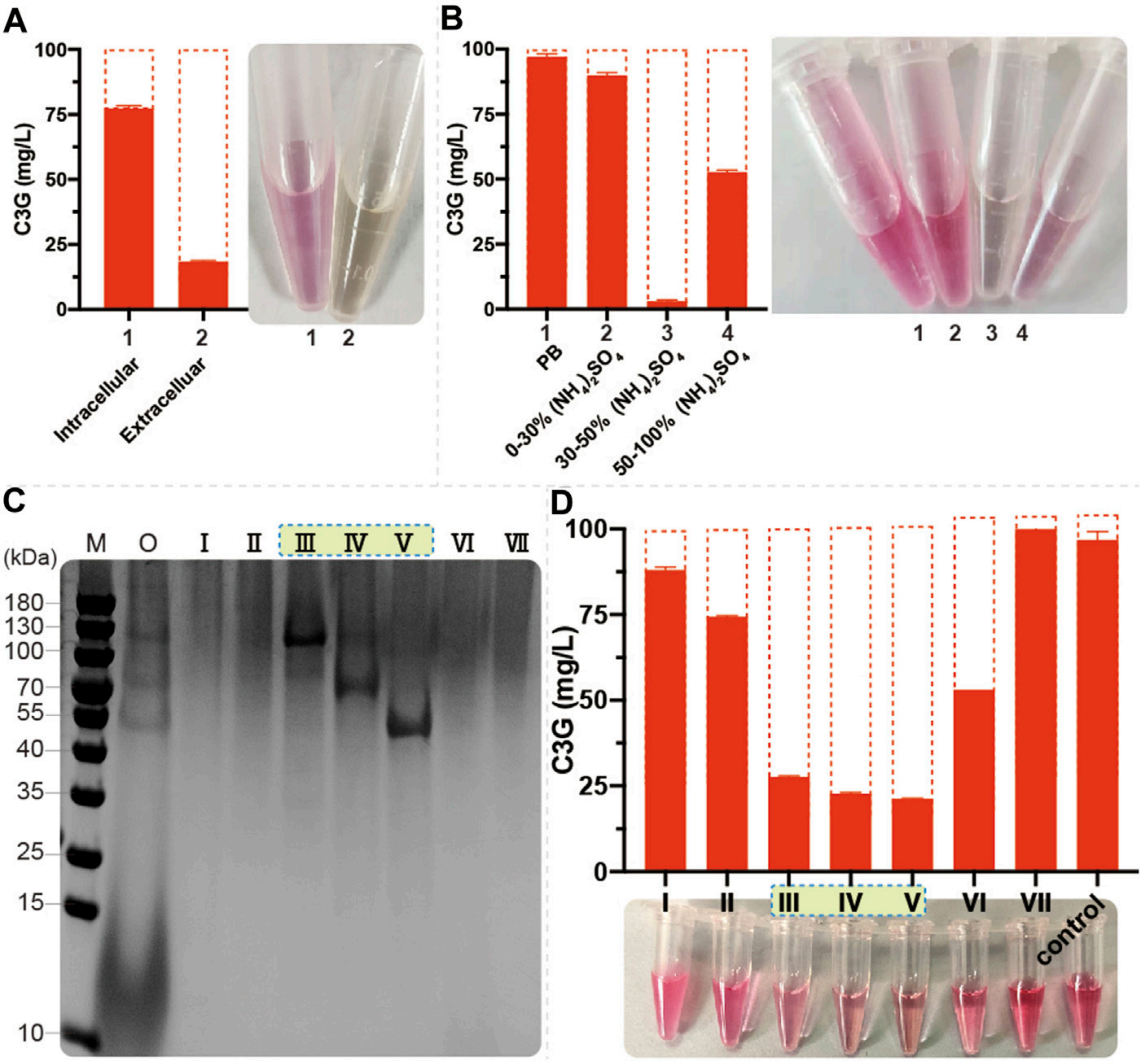
Identification of endogenous anthocyanin-degrading enzymes **(A)** 1 ml of fermentation broth (with YPD medium) of CENPK2-1D was centrifuged at 6000g and 30°C for 1 min; the supernatant was extracellular proteins. The cell pellet was washed twice with 2 ml of 20 mmol/L PB (pH = 5.0, 4°C). The intracellular proteins were obtained by cell disruption with 1 ml of 20 mm PB and 0.1-mm glass beads in FastPrep-24 5^G^. Then, 100 mg/L C3G was added to extracellular and intracellular proteins at 220 rpm and 30°C for 12-h incubation. An equal volume of acidified methanol as the chromogenic reagent was added to the sample **(B)** Then, 0%–30%, 30%–50%, and 50–100% protein components were obtained during ammonium sulfate precipitation; 100 mg/L C3G was added for 12-h incubation at 220 rpm and 30°C. An equal volume of acidified methanol as the chromogenic reagent was added to the sample **(C)** M: marker; O: 30–50% protein component; Ⅰ–Ⅶ: seven elution signal peaks **(D)** 100 mg/L C3G was added to Ⅰ–Ⅶ for 12-h incubation at 220 rpm and 30°C. An equal volume of acidified methanol as the chromogenic reagent was added to the sample.

To further investigate the phenomenon of anthocyanin degradation, 100 mg/L C3G was added to the SC and YPD medium using CENPK2-1D. The C3G degraded much faster in nutrient-rich YPD medium than in SC medium (Supplementary Figure S1A), suggesting that the anthocyanins underwent significant enzymatic degradation in *S. cerevisiae*. The 24-h fermentation broth (with YPD medium) of CENPK2-1D was centrifuged to collect the extracellular proteins, and the cells were fragmented to collect the intracellular proteins (with 20 mmol/L PB, pH = 5.0 buffer). Furthermore, 100 mg/L C3G was added to extracellular and intracellular proteins for 12 h (220 rpm, 30°C). The results showed that the degradative protein mainly existed extracellularly (Figure 4A). According to the protocol of ammonium sulfate precipitation, the ammonium sulfate precipitation protein components of 0%–30%, 30%–50%, and 50–100% were obtained, and the 100 mg/L C3G was added for 12-h incubation at 220 rpm and 30°C. The result showed that the degradative protein mainly existed in 30–50% of the protein components (Figure 4B).

Size-exclusion chromatography was performed to separate 0–50% of protein components, and seven elution signal peaks were collected. The proteins corresponding to Ⅲ, Ⅳ, and Ⅴ protein bands had a noticeable degradation effect on anthocyanins (Figures 4C,D). The anthocyanin degradation ratio of Ⅲ, Ⅳ, and Ⅴ was 72.4, 77.3, and 78.7%, respectively. Then, using the protein profiles, four potential anthocyanin-degrading enzymes, namely, *SCW2*, *SIM1*, *SCW4*, and *EXG1* (Table 2) were selected by protein profile identification. Moreover, *SPR1* and *YIR007W* were reported to be efficient β-glucosidases (Schmidt et al., 2011) considered to be potential anthocyanin-degrading enzymes.

**TABLE 2:** Potential anthocyanin-degrading enzymes.

Number	Matching protein	Sequence coverage (%)	Score	Description
Ⅲ-1	*SCW2*	6	852	Glycosidases and chitinase
Ⅲ-2	*SIM1*	22	191	Probable secreted beta-glucosidase
Ⅳ	*SCW4*	52	2,579	Probable family 17 glucosidase
Ⅴ	*EXG1*	81	7,691	Glucan 1,3-beta-glucosidase

The endogenous genes of yeast are generally shown in italics.

### Knockout of anthocyanin-degrading enzymes

**FIGURE 5:**
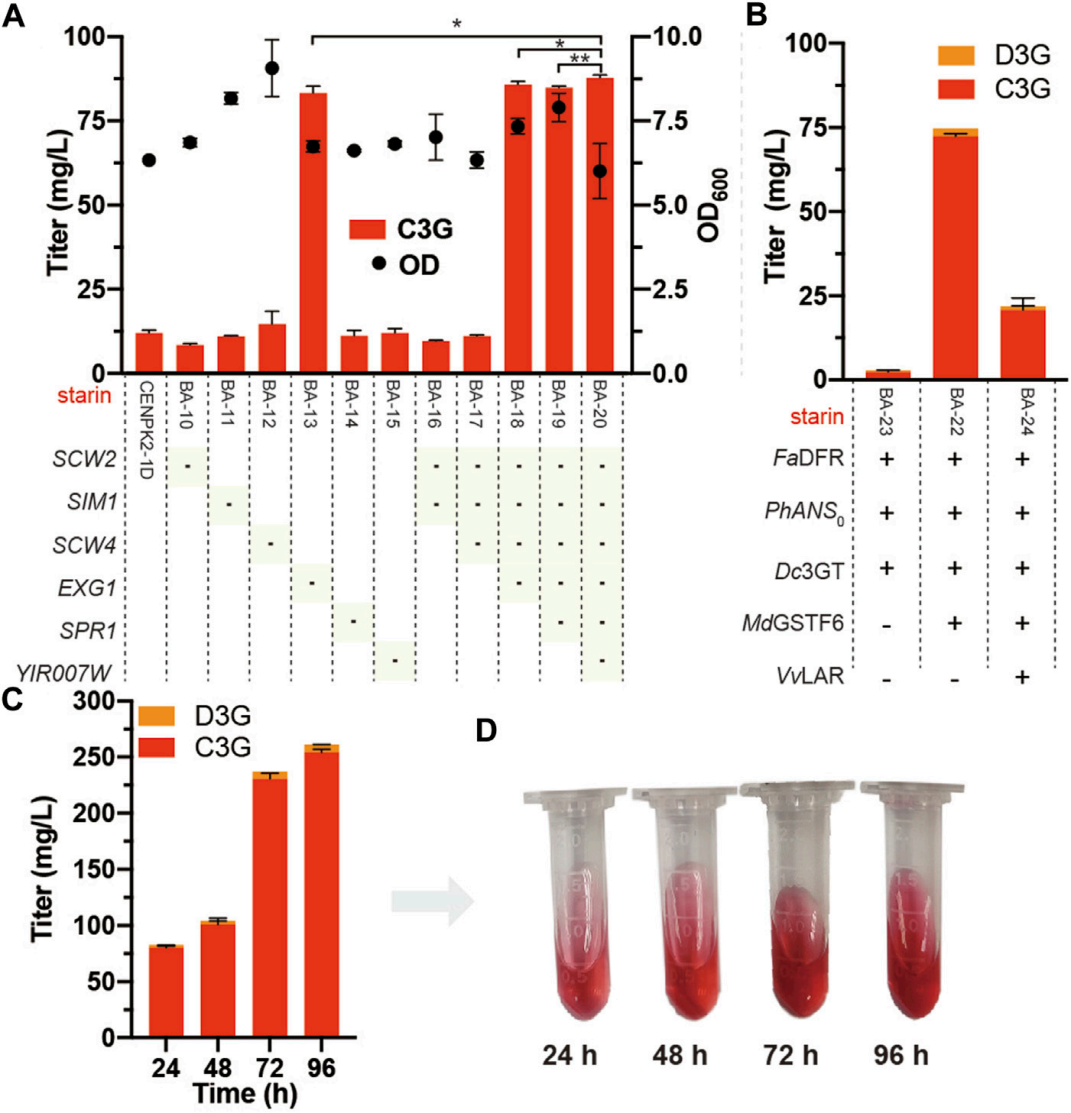
Knockout of anthocyanin-degrading enzymes **(A)** Marker of “**-**” indicates that the corresponding gene was knocked out in the CENPK2-1D. Student’s *t* test (one-tailed; two-sample unequal variance; ∗*p* < 0.05, ∗∗*p* < 0.01, ∗∗∗*p* < 0.001) was performed by Origin 2018 **(B)** *Fa*DFR, *Ph*ANS_0_, and *Md*GSTF6 were promoted by P_TDH1_, P_GAL10_, and P_INO1_ in the BA-21 strain with the pY26-TEF-GPD supplemented with 500 mg/L dihydroflavonols for 24-h cultivation, respectively. *Vv*LAR was connected to the N-terminal of *Fa*DFR with a flexible (GGGGS)_2_ linker (CTA​CCG​CCA​CCG​CCG​CTA​CCG​CCA​CCG​CC) and promoted by P_TDH1_ **(C)** 96-h fermentation of the BA-22 strain; 500 mg/L dihydroflavonols were added after 0 h, 1,000 mg/L dihydroflavonols were added after 24 h, and 500 mg/L dihydroflavonols were added after 48 h **(D)** Fermentation broth corresponding to different times. Equal volume of acidified methanol as the chromogenic reagent was added to the sample.

The six potential anthocyanin-degrading enzymes (*SCW2*, *SIM1*, *SCW4*, *EXG1*, *SPR1*, and *YIR007W*) were knocked out in the strain CENPK2-1D, resulting in strains BA-10, BA-11, BA-12, BA-13, BA-14, BA-15, and BA-16. The anthocyanin degradation ratio of BA-13 showed that disrupting *EXG1* significantly reduced the degradation rate from 88.0 to 17.3%. Thus, higher anthocyanin production could be achieved by knocking out *EXG1* (Figure 5A). At the lowest anthocyanin degradation ratio (12.2%), the BA-20 strain was integrated with *Dc*3GT in the *GAL80* site, resulting in BA-21. As shown in Figure 5B, although the anthocyanin-degrading enzymes knocked out the BA-21 strain, anthocyanins could not be synthesized effectively; the titer of anthocyanins was 2.9 mg/L (2.4 mg/L C3G and 0.5 mg/L D3G). The BA-21 strain further expressed *Md*GSTF6 (the crucial enzyme for anthocyanin biosynthesis in *S. cerevisiae*), and 74.7 mg/L anthocyanins (72.3 mg/L C3G and 2.4 mg/L D3G) were obtained.

In addition, flavan-3-ols are the precursors of anthocyanins obtained by the introduction of leucoanthocyanidin reductase (LAR) into the anthocyanin biosynthetic pathway (Figure 1). According to our previous study (Sun et al., 2021), *Vv*LAR was connected to the *Fa*DFR N-terminal with a flexible (GGGGS) 2 linker (CTA​CCG​CCA​CCG​CCG​CTA​CCG​CCA​CCG​CC), and the output of flavan-3-ols increased sevenfold. However, the introduction of *Vv*LAR reduced the production of anthocyanins; out of our expectation, only 21.9 mg/L anthocyanins (20.8 mg/L C3G and 1.1 mg/L D3G) were obtained (Figure 5B). When supplemented with 2.0 g/L dihydroflavonols, the accumulation of anthocyanins reached 261.6 mg/L (254.5 mg/L C3G and 7.1 mg/L D3G) by BA-22 (BA-21 harboring the pY26-P_TDH1_-*Fa*DFR-T_GAA1_-P_GAL10_-*Ph*ANS_0_-T_ALT1_-P_INO1_-*Md*GSTF6-T_CYC1_ plasmid) (Figures 5C,D).

### *De novo* biosynthesis of anthocyanins

**FIGURE 6:**
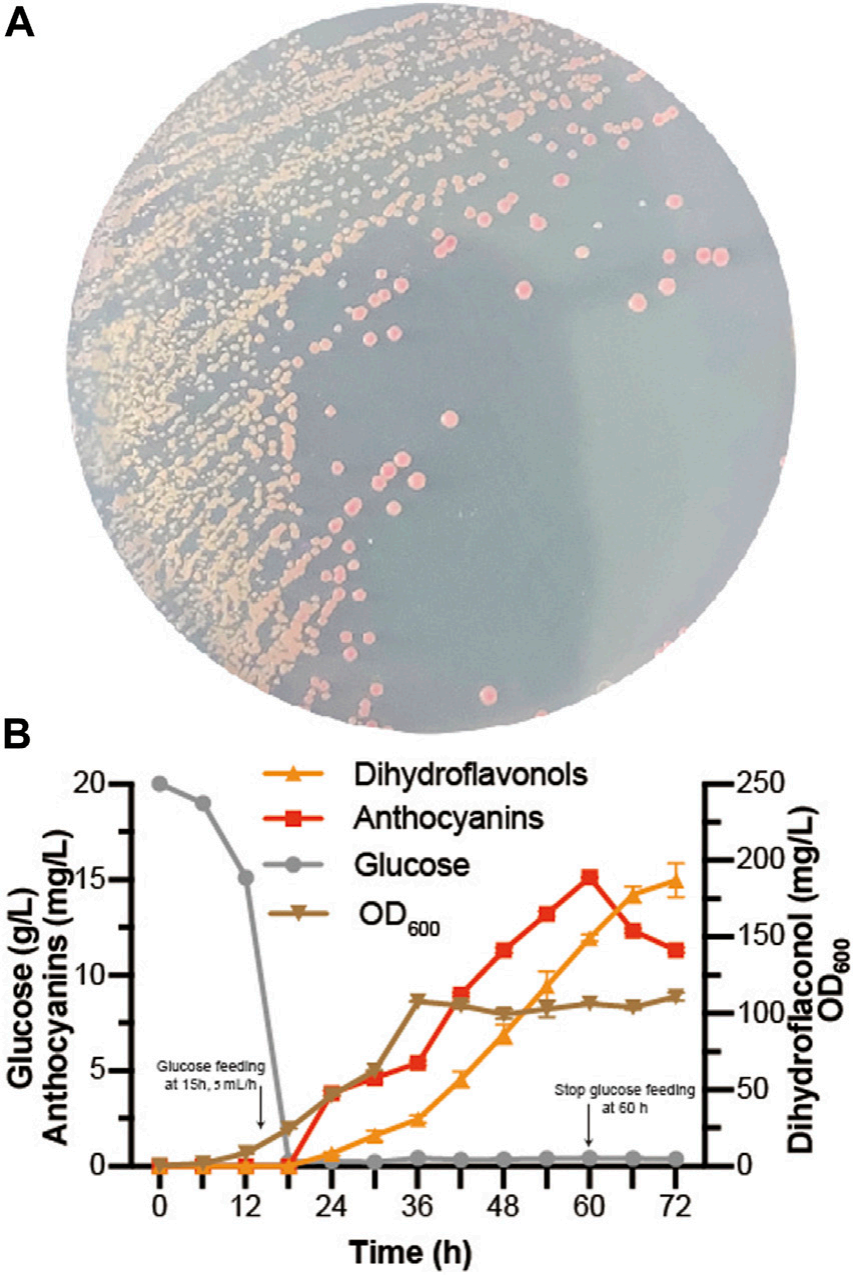
*De novo* biosynthesis of anthocyanins **(A)** BA-32 strain was grown for 4 days in the SC medium plate **(B)** Dihydroflavonols, including dihydrokaempferol, dihydroquercetin, and dihydromyricetin; anthocyanins, including pelargonidin-3-*O*-glucoside, cyanidin-3-*O*-glucoside, delphinidin-3-*O*-glucoside, pelargonidin, cyaniding, and delphinidin. The fed-batch fermentation was performed with a 5-L bioreactor containing 2.5 L of YPD. The dissolved oxygen was maintained at 10% (degree of saturation) with the agitation ranging from 250 to 600 rpm. The glucose was fed after 15 h at the rate of 5 ml/h and stopped at 60 h. After 24 h, 0.05 mmol/L α-ketoglutaric acid, 0.5 mmol/L ascorbic acid, and 0.5 mmol/L FeSO_4_ were added individually to the bioreactor.

At present, some progress has been achieved in the heterologous biosynthesis of dihydroflavonols in *S. cerevisiae* (Li G. et al., 2020; Gao et al., 2020), laying the foundation for *de novo* biosynthesis of anthocyanins. As *EXG1* was proven to be the main anthocyanin-degrading enzyme, *Dc*3GT was integrated into the *EXG1* site of BM-31 (Li G. et al., 2020), resulting in BA-31. Then, the pY26-P_TDH1_-*Fa*DFR-T_GAA1_-P_GAL10_-*Ph*ANS_0_-T_ALT1_-P_INO1_-*Md*GSTF6-T_CYC1_ plasmid was transformed into BA-31, resulting in BA-32. Red colonies grew on the SC medium plate because of successful expression of the anthocyanin *de novo* biosynthesis pathway in *S. cerevisiae* (Figure 6A). Fed-batch fermentation was performed in the 5-L bioreactor for 72 h. The results are shown in Figure 6B. A large amount of dihydroflavonols accumulated, containing 11.3 mg/L dihydrokaempferol, 138.5 mg/L dihydroquercetin, and 37.1 mg/L dihydromyricetin. The highest titer of anthocyanins was obtained in 60 h (15.1 mg/L, containing 8.0 mg/L C3G, 3.0 mg/L CYA, 3.5 mg/L D3G, and 0.7 mg/L DEL), after which the titer of anthocyanins decreased gradually.

## Discussion

The production of anthocyanins with microorganisms by metabolic engineering is meaningful for sustainable development, whereas the large-scale production of anthocyanins is still difficult (Li J. et al., 2020). The expensive flavan-3-ols (afzelechin and catechin) are usually the substrates for producing anthocyanins by ANS and 3 GT in prokaryotes. In this study, anthocyanins were successfully obtained from dihydroflavonols. A high titer of anthocyanins was harvested, reaching 261.6 mg/L, through the introduction of *Md*GSTF6 and the knockout of anthocyanin-degrading enzymes. Finally, by assembling the anthocyanin *de novo* biosynthetic pathway, 15.1 mg/L anthocyanins were obtained.

In plant cells, the stable storage of anthocyanins is inseparable from the transportation of anthocyanins; however, the mechanism is unclear (Behrens et al., 2019). *Md*GSTF6 is an anthocyanin transporter involved in transporting anthocyanins from the cytoplasm to the vacuole in the plant cell (Jiang et al., 2019). However, it did not transport anthocyanins to the vacuole of *S. cerevisiae* because more than 90% of anthocyanins were transported to the extracellular environment. Actually, as polyphenols, many flavonoids, including anthocyanins, would be toxic to cells during intracellular accumulation. In *E. coli*, the production of anthocyanins could be improved by enhancing the secretion of anthocyanins (Lim et al., 2015). The transport of target products is of positive significance for their accumulation. In this aspect, *S. cerevisiae* has obvious advantages over *E. coli* for anthocyanin production. In microorganisms, 2*R*,4*R*-flavan-3,3,4-triol, in the production of leucoanthocyanidin by ANS, prefers dehydration to form double bonds at C3–C4 to C2–C3 (Zhang et al., 2019). The resulting production leads to the formation of flavonols after a series of spontaneous reactions and the catalysis of ANS. (Figure 2B). However, under the action of *Md*GSTF6, the 2*R*,4*R*-flavan 3,3,4-triol was likely to form double bonds at C2–C3, which was beneficial to the formation of anthocyanins.

Although the biosynthesis of anthocyanins has been extensively studied (Morita et al., 2014), the mechanism of anthocyanin degradation by *S. cerevisiae* remains unclear (Pojer et al., 2013). *EXG1* was found to be the main anthocyanin-degrading enzyme in this study. *EXG1* was reported to be the flavonoid glycoside hydrolase with significant hydrolysis of anthocyanidins (Schmidt et al., 2011) and C-7 flavonoid glycosides (Lyu et al., 2020). Through the knockout of *EXG1*, the titer of anthocyanins was approximately increased by 100% in the case of co-expressing *Md*GSTF6. However, an improvement in anthocyanin production by deleting *EXG1* was not detected in the absence of *Md*GSTF6 (Figure 5B). *EXG1* did not have a strong hydrolytic effect on the 3-position of flavonoid glycoside because trace amounts of quercetin (QUE) were detected during the co-culture of Q3G with *S. cerevisiae* (Supplementary Figure S1). Aromatic compound lyases exist in many microorganisms (Patel et al., 2017; Barbosa et al., 2019). Kallscheuer et al. prevented the degradation of phenylpropanoids in *Corynebacterium glutamicum* by deleting related gene clusters for constructing a platform strain of overproducing stilbenes and (2S)-flavanones (Kallscheuer et al., 2016). The degradation of C3G might be due to the cleavage of *EXG1*. In fact, the speculative C3G degradation products were reported previously (Barbosa et al., 2019), and protocatechuic acid and phloroglucinol aldehyde were detected by LC-MS (Supplementary Figure S2).

ANS is the key as well as the multifunctional enzyme in the anthocyanin biosynthetic pathway. As mentioned earlier, ANS has the same function as FLS that can convert dihydroflavonols into flavonols. In addition, ANS also had the function of F3H belonging to 2ODD, which could also convert dihydroflavonoids into corresponding dihydroflavonols. When naringenin was supplemented to the *S. cerevisiae* expressing ANS, dihydrokaempferol and kaempferol were detected simultaneously. The promiscuity of ANS could lead to the accumulation of the by-products flavonol-3-*O*-glucosides, such as Q3G and M3G. Fortunately, in the presence of *Md*GSTF6, the titer of anthocyanins was increased by 13-fold, while the flavonol-3-*O*-glucoside was decreased by sevenfold. In contrast to the prokaryotes (*E. coli*, *C. glutamicum*, and *L. lactis*), *S. cerevisiae* hardly produced anthocyanins with the flavan-3-ols as the substrate when expressing ANS and 3GT in the *EXG1* knockout strain, which was consistent with a previous study (Eichenberger et al., 2018). When co-expressed with *Md*GSTF6 and supplemented with 500 mg/L catechin (Supplementary Figure S5), 2.3 mg/L C3G was detected, which was increased 15-fold compared to the control group (without *Md*GSTF6). This suggests that *Md*GSTF6 also has the potential ability to assist the conversion of flavan-3-ols to anthocyanins.

To further increase the production of anthocyanins, some problems should be resolved in the future. The anthocyanins tend to degrade due to the instability of their molecular structure (Coutinho et al., 2015). pH is one of the main factors affecting the stabilization of anthocyanins. Generally, anthocyanins are prone to degradation in a neutral or alkaline environment (Coutinho et al., 2015), which limits their application in complex environments. The stability of anthocyanins could be improved by some modifications, such as introducing acetyl and methyl groups (Zhao et al., 2017). The supply of UDP-glucose was an important factor in anthocyanin production (Lim et al., 2015; Zha et al., 2018). However, it was not suitable to add it to the medium as a substrate, owing to its excessive values. Rewiring UDP-glucose metabolism in *S. cerevisiae* should be an effective method (Feng et al., 2020). In addition, directed evolution is an important technology to improve the catalytic efficiency of key enzymes. Also, the intensity of red colonies caused by anthocyanins is a promising high-throughput screening indicator, which makes it effective to select high-catalytic activity mutants.

## Data Availability

The datasets presented in this study can be found in online repositories. The names of the repository/repositories and accession number(s) can be found in the article/Supplementary Material.
