# Supplementary material for: Efficient production of anthocyanins in Saccharomyces cerevisiae by introducing anthocyanin transporter and knocking out endogenous degrading enzymes
Source: Front Bioeng Biotechnol. 2022 Aug 19;10:899182. doi: 10.3389/fbioe.2022.899182 (PMC9437251; doi:10.3389/fbioe.2022.899182)
Supplement: Supplementary file 1 [file DataSheet1.docx]

**Table S1 The genes used in this study.**

| **Gene** | **Source** | **Accession number** |
| --- | --- | --- |
| *Fa*DFR | *Fragaria x ananassa* | KC894055.1 |
| *D*ANS | *Dahlia pinnata* | AB591830.1 |
| *Gh*ANS | *Gerbera hybrid* | AY997840.2 |
| *Gh*ANS_0_ | *Gerbera hybrid* | AY997840.2 |
| *Lr*ANS | *Lycium ruthenicum* | KY287796.1 |
| *Md*ANS | *Malus domestica* | AF117269.1 |
| *Ts*ANS | *Tricyrtis sp. Shinonome* | LC209106 |
| *Vv*ANS | *Vitis vinifera* | EF192468.1 |
| *Bo*ANS | *Brassica oleraceavar. capitata* | AY228485.1 |
| *Cs*ANS | *Citrus sinensis* | XM_025097974.1 |
| *Gm*ANS | *Glycine max* | EU334548.1 |
| *Mi*ANS | *Matthiola incana* | AF026058 |
| *Ph*ANS_0_ | *Petunia x hybrida]* | ([Zha et al., 2018](#_ENREF_37)) |
| *At*3GT | *Arabidopsis thaliana* | KJ138682.1 |
| *At*3GT_0_ | *Arabidopsis thaliana* | KJ138682.1 |
| *Dc*3GT | *Dianthus caryophyllus* | BAD52003.1 |
| *Fa*3GT | *Fragaria ananassa* | AAU12366.1 |
| *At*TT12 | *Arabidopsis thaliana* | NP_191462.1 |
| *At*GFS9 | *Arabidopsis thaliana* | NP_566837.1 |
| *Md*GSTF6 | *Malus domestica* | NP_001315851.1 |
| *Vv*LAR | *Vitis vinifera* | CAI26310.1 |

The subscript 0 represents the codon of the genes original from plant and the other genes were codon-optimized base on *S. cerevisiae*. The sequence of genes used in this work were list in Supplementary materials 2.

**Table S2 The main plasmids used in this study**

| Number | Plasmid |
| --- | --- |
| 1 | pY26-P_TDH1_-*Fa*DFR-T_GAA1_-P_GAL10_-*Md*ANS-T_ALT1_ |
| 2 | pY26-P_TDH1_-*Fa*DFR-T_GAA1_-P_GAL10_-*Md*ANS_0_-T_ALT1_-P_INO1_-*At*TT12-T_CYC1_ |
| 3 | pY26-P_TDH1_-*Fa*DFR-T_GAA1_-P_GAL10_-*Md*ANS_0_-T_ALT1_-P_INO1_-*At*GSF9-T_CYC1_ |
| 4 | pY26-P_TDH1_-*Fa*DFR-T_GAA1_-P_GAL10_-*Md*ANS_0_-T_ALT1_-P_INO1_-*Md*GSTF6-T_CYC1_ |
| 5 | pY26-P_TDH1_-*Fa*DFR-T_GAA1_-P_GAL10_-*Ph*ANS_0_-T_ALT1_-P_INO1_-*Md*GSTF6-T_CYC1_ |
| 6 | pY26-P_TDH1_-*Fa*DFR-T_GAA1_-P_GAL10_-*Dp*ANS-T_ALT1_-P_INO1_-*Md*GSTF6-T_CYC1_ |
| 7 | pY26-P_TDH1_-*Fa*DFR-T_GAA1_-P_GAL10_-*Gh*ANS-T_ALT1_-P_INO1_-*Md*GSTF6-T_CYC1_ |
| 8 | pY26-P_TDH1_-*Fa*DFR-T_GAA1_-P_GAL10_-G*h*ANS_0_-T_ALT1_-P_INO1_-*Md*GSTF6-T_CYC1_ |
| 9 | pY26-P_TDH1_-*Fa*DFR-T_GAA1_-P_GAL10_-*Lr*ANS-T_ALT1_-P_INO1_-*Md*GSTF6-T_CYC1_ |
| 10 | pY26-P_TDH1_-*Fa*DFR-T_GAA1_-P_GAL10_-*Md*ANS-T_ALT1_-P_INO1_-*Md*GSTF6-T_CYC1_ |
| 11 | pY26-P_TDH1_-*Fa*DFR-T_GAA1_-P_GAL10_-*Ts*ANS-T_ALT1_-P_INO1_-*Md*GSTF6-T_CYC1_ |
| 12 | pY26-P_TDH1_-*Fa*DFR-T_GAA1_-P_GAL10_-*Vv*ANS-T_ALT1_-P_INO1_-*Md*GSTF6-T_CYC1_ |
| 13 | pY26-P_TDH1_-*Fa*DFR-T_GAA1_-P_GAL10_-*Bc*ANS-T_ALT1_-P_INO1_-*Md*GSTF6-T_CYC1_ |
| 14 | pY26-P_TDH1_-*Fa*DFR-T_GAA1_-P_GAL10_-*Cs*ANS-T_ALT1_-P_INO1_-*Md*GSTF6-T_CYC1_ |
| 15 | pY26-P_TDH1_-*Fa*DFR-T_GAA1_-P_GAL10_-*Gm*ANS-T_ALT1_-P_INO1_-*Md*GSTF6-T_CYC1_ |
| 16 | pY26-P_TDH1_-*Fa*DFR-T_GAA1_-P_GAL10_-*Mi*ANS-T_ALT1_-P_INO1_-*Md*GSTF6-T_CYC1_ |
| 17 | pY26-P_TDH1_-*Fa*DFR-(GGGGS)2-*Vv*LAR-T_GAA1_-P_GAL10_-*Ts*ANS-T_ALT1_-P_INO1_-*Md*GSTF6-T_CYC1_ |
| 18 | pY26-P_GAL10_-*Ph*ANS_0_-T_ALT1_ |
| 19 | pY26-P_GAL10_-*Ph*ANS_0_-T_ALT1_-P_INO1_-*Md*GSTF6-T_CYC1_ |
| 20 | pY26-P_TDH1_-*Fa*DFR-T_GAA1_-P_GAL10_-*Ph*ANS-T_ALT1_ |

**Table S3 The 20 nt guide sequence of endogenous gens of *S*. *cerevisiae.***

| Gene | 20 nt (5’-3’) |
| --- | --- |
| *GAL80* | gcaaaagaattaatatctca |
| *SCW2* | aacttttgcacaaactttat |
| *SIM1* | gccactgcctcaacatctca |
| *SCW4* | agcagcagacgatgttgtac |
| *EXG1* | cattactccatctttgttcg |
| *SPR1* | ttattggaatcgaattgctc |
| *YIR007W* | cagttgctctcaaacaactc |


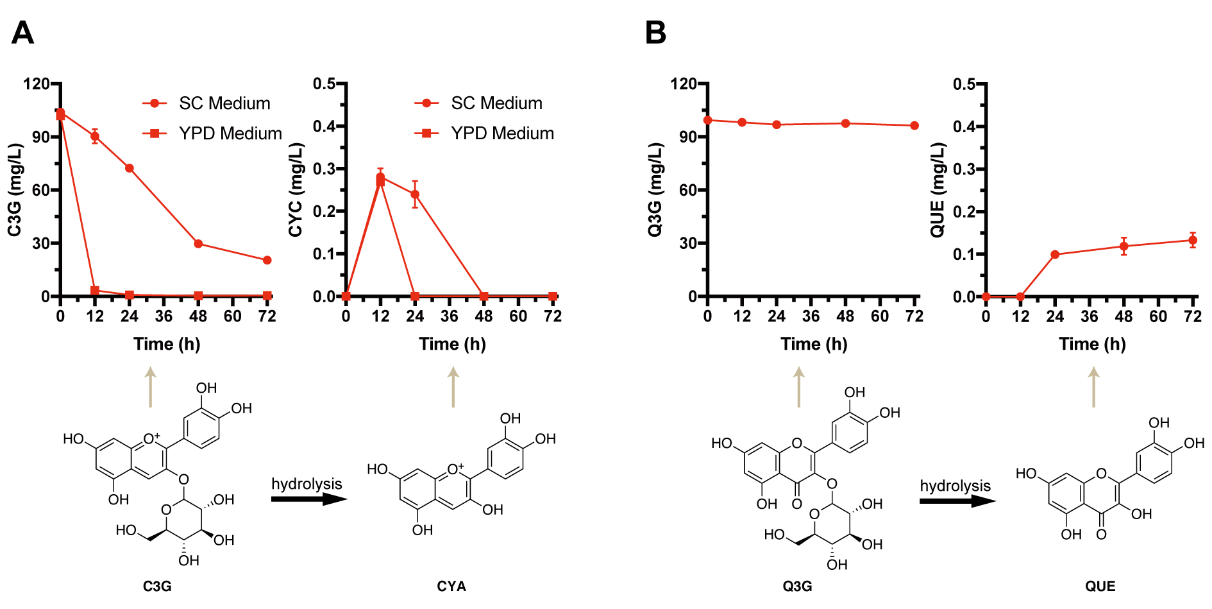


**Figure S1 The degradation of C3G and Q3G in *S. cerevisiae*.**


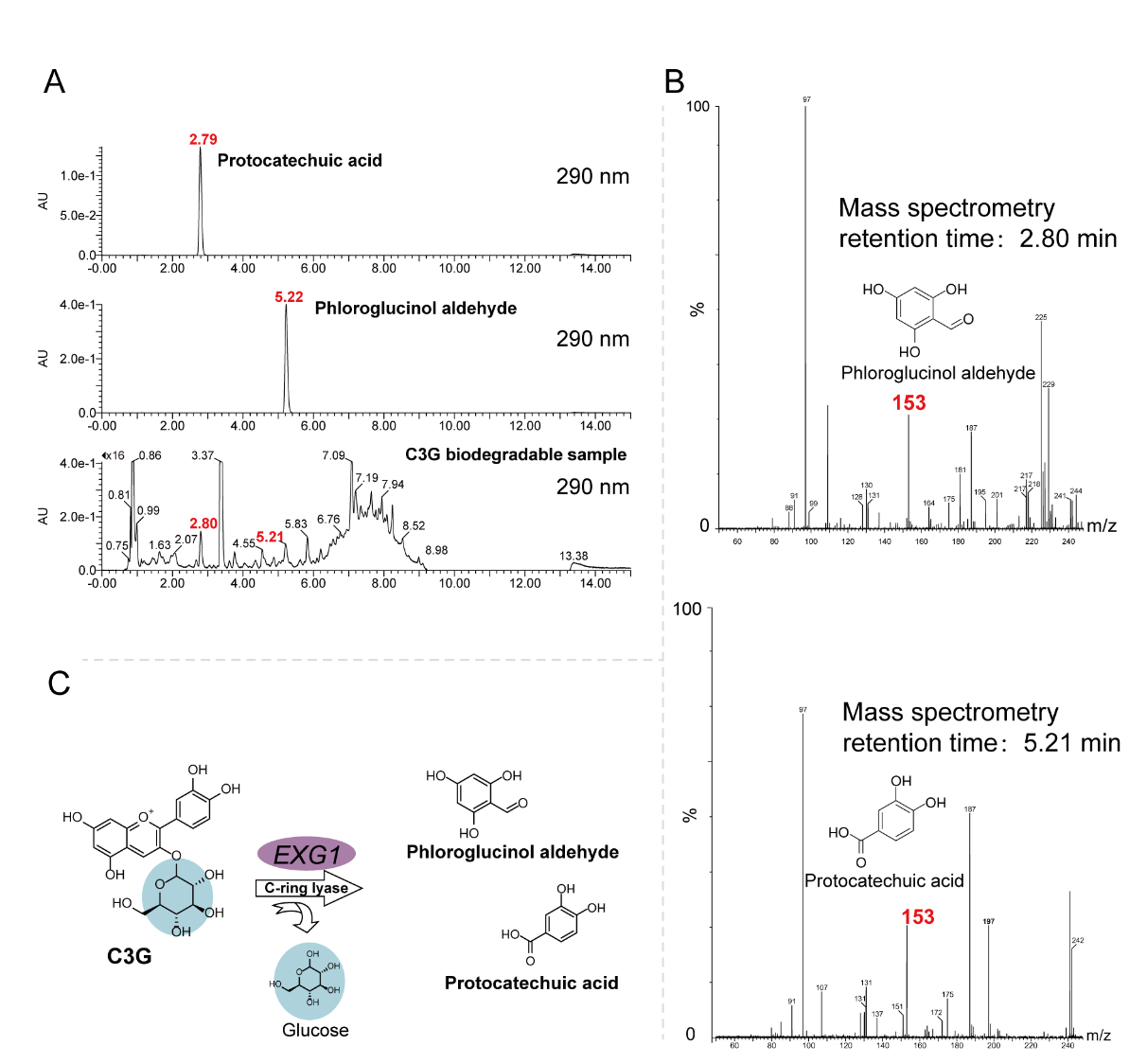


**Figure S2 Qualitative analysis of anthocyanin degradation products.**


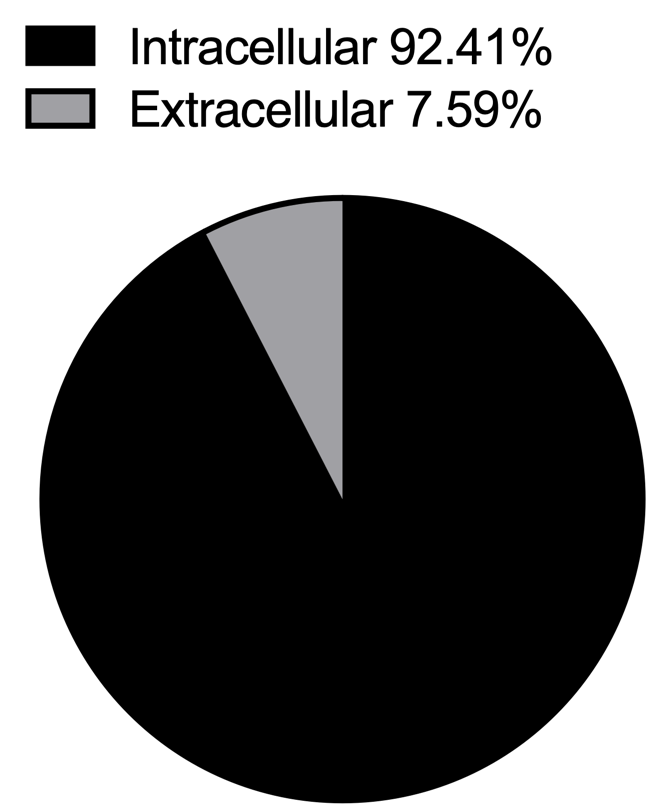


**Figure S3 Distribution of anthocyanins in *S. cerevisiae*.**


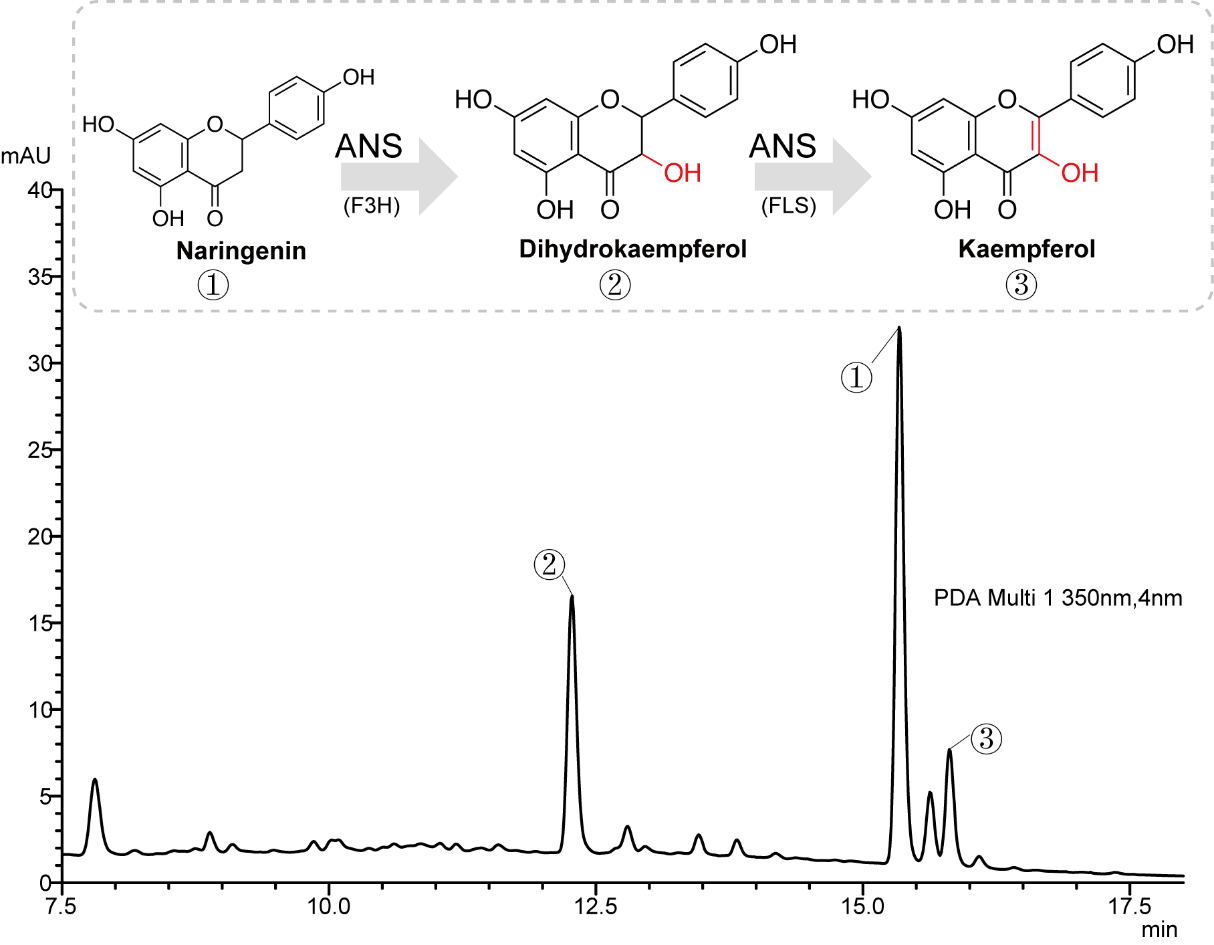


**Figure S4 The multifunction of ANS.**


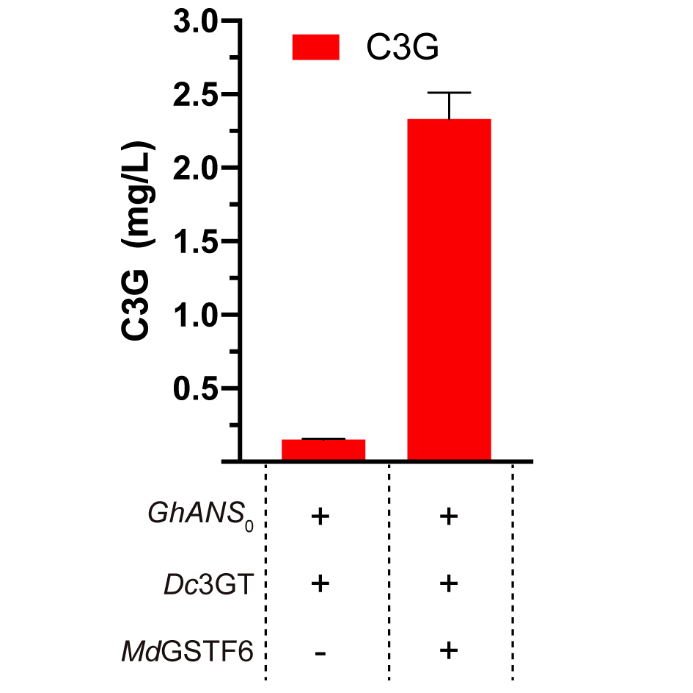


**Figure S5 The utilization of catechins in *S. cerevisiae*.**
